# Unveiling diverse solitons in the quintic perturbed Gerdjikov-Ivanov model via a modified extended mapping method

**DOI:** 10.1038/s41598-025-97981-6

**Published:** 2025-05-07

**Authors:** Hisham H. Hussein, Hamdy M. Ahmed, Shaimaa A. Kandil, Wassim Alexan

**Affiliations:** 1 School of Mathematical and Computational Sciences, University of Prince Edward Island (UPEI), hosted by Universities of Canada in Egypt, Cairo, 11835 Egypt; 2 Department of Physics and Engineering Mathematics, Higher Institute of Engineering, El Shorouk Academy, El-Shorouk City, Cairo Egypt; 3 Department of Electrical Power and Machine Engineering, Faculty of Engineering, Helwan University, Cairo, Egypt; 4 Faculty of Information Engineering and Technology, The German University in Cairo (GUC), Cairo, Egypt; 5 Department of Mathematics, The German International University (GIU), New Administrative Capital, Cairo, Egypt

**Keywords:** Applied mathematics, Computational science

## Abstract

The quintic perturbed Gerdjikov–Ivanov equation, a non-linear model in optics and quantum field theory, describes the propagation of optical pulses in nonlinear media with quintic nonlinearity and perturbation effects. This study aims to derive exact traveling wave solutions for the quintic perturbed Gerdjikov–Ivanov equation using the modified extended mapping method. The method efficiently generates a broad spectrum of solutions, including bright, dark, periodic, singular periodic, hyperbolic, plane, Weierstrass, and Jacobi elliptic forms, extending the known solution space. Compared to previous techniques, such as the generalized exponential rational function and Kudryashov’s methods, the modified extended mapping method provides a more diverse set of analytical solutions with improved computational efficiency. Graphical representations using Mathematica illustrate the physical properties and stability of these solutions, confirming their relevance to optical communication and nonlinear wave phenomena. This work advances the understanding of soliton dynamics in nonlinear media and demonstrates the potential of the modified EM method in solving complex non-linear partial differential equations.

## Introduction

Soliton pulse propagation is a fundamental concept in understanding various models within optics and metamaterials, particularly those characterized by intensity-dependent phase variations. These models are essential in developing technologies such as optical pulse compressors, fiber-optic amplifiers, and communication systems. The distinctive feature of solitons—their ability to maintain shape during propagation—has garnered significant scientific interest. As a result, considerable efforts were devoted to obtaining analytical solutions for non-linear partial differential equations (NPDEs) using advanced computational methods^1–8^. While extensively studied models like the non-linear Schrödinger equation (NLSE) have paved the way, other equations also received attention in recent literature. Those include the complex Ginzburg-Landau^9^, Sasa-Satsuma^10^, Lakshmanan-Porsezian-Daniel^11^, and longitudinal wave equation within the context of a Magneto-Electro-Elastic annular bar^12^.

To meet the demands of modern telecommunication systems, this research focuses on the pGI equation, a crucial non-linear evolution equation^13^. The Gerdjikov-Ivanov (GI) equation, a cornerstone model in the study of non-linear optical fibers, especially in crystal fibers of photonic nature, has been extensively studied due to its intricate dynamics. Deriving exact analytical solutions for such partial differential equations remains a formidable challenge, often requiring numerical or approximate methods. Nonetheless, exact solutions are inherently preferred for their directness and unrestricted applicability. Previous research has explored the QpGI equation through various approaches: the generalized exponential rational function method^14^; the hyperbolic extended function and generalized Kudryashov’s methods^15^; the Nucci reduction method^16^; the conformable derivative has played a key role in exploring chirp soliton solutions with hyperbolic functional terms^17^; and the Riccati-Bernoulli sub-ODE method, combined with the Bäcklund transformation^18^. Collectively, these methodologies have significantly advanced our knowledge of the GI equation and its implications for non-linear phenomena.

In recent times, a plethora of mathematical techniques have been explored for constructing solutions to non-linear partial differential equations (NPDEs). Prominent among these are the Riemann-Hilbert method^19,20^, the Jacobi elliptic function method^21,22^, Lie symmetric analysis^23,24^, the auxiliary equation method^25,26^, the Sine-Gordon expansion method^27,28^, the tan-cot function method^29^, the Sardar-sub equation technique^30^, the simple equation method^31^, the modified simple equation method^32^, the first integral method^33,34^, Hirota’s bilinear method^35,36^, the homogeneous balance method^37,38^, the Darboux-Like transformation method^39^, and the $\:exp(-\:\phi\:(\xi\:)$) method^40^.

The modified EM method has emerged as a powerful tool for uncovering diverse soliton solutions in various NPDEs. Studies have successfully employed the EM method to find bright, dark, and singular soliton solutions in complex systems like coupled non-linear Maccari systems^41^ and the (4 + 1)-dimensional Davey–Stewartson–Kadomtsev–Petviashvili equation^42^. It has also been instrumental in discovering novel solutions beyond solitons, including singular periodic, rational, exponential, and Weierstrass elliptic forms in models like the concatenation model^43^ and the KP-BBME water wave model [44]. The method’s applicability extends to other contexts, as demonstrated in^45^ where it retrieved solutions for the Gilson-Pickering equation used in plasma physics. The applied method outperforms previous techniques by generating a more diverse set of exact solutions, extending beyond those mentioned before.

This research work treats the dynamics of soliton propagation through the (1 + 1)-dimensional QpGI equation. Unlike the commonly studied cubic non-linear Schrödinger equation, the QpGI equation incorporates quintic non-linearity. To carry out an investigation of the soliton solutions of this model, we employ the modified EM method. The derived solutions are visually represented through 2D and 3D and density plots to interpret their non-linear physical behavior. A comprehensive analysis of the obtained results, including their classification, is presented in subsequent sections, along with concluding remarks.

## Description of the adopted QpGI model

The key contribution of this article is to employ a novel method in the derivation of various novel solutions to a variant form of an QpGI. In particular, the QpGI equation is given as in^13^ by:

1 $$i{{\mathcal{A}}_t} + a~{{\mathcal{A}}_{xx}} + b~\left| {{{\mathcal{A}}^4}} \right|{\mathcal{A}} + i~c~{{\mathcal{A}}^2}{\mathcal{A}}_x^* = i\left[ {{a_1}{{\mathcal{A}}_x} + {b_1}{{\left( {{\mathcal{A}}{{\left| {\mathcal{A}} \right|}^{2n}}} \right)}_x} + {c_1}{{\left( {{{\left| {\mathcal{A}} \right|}^{2n}}} \right)}_x}{\mathcal{A}}} \right],$$

given that $\:\mathcal{A}\left(x,t\right)$ is the complex wave function that, when $\:x$ and $\:t\:$are the spatial and temporal variables in turn, acts as the complex-valued wave structure representing optical solitons in polarization-preserving fibers. The first term, $\:{\mathcal{A}}_{t}$, denotes the solitons’ linear temporal progression. The group velocity dispersion is denoted by $\:{\mathcal{A}}_{xx}$, the non-linear dispersion with coefficients $\:a,\:b,$ and $\:c$, respectively, is represented by $\:{\mathcal{A}}^{2}{\mathcal{A}}_{x}^{{\ast}}$, and the model’s current quintic non-linearity is shown by $\:\left|{\mathcal{A}}^{4}\right|\mathcal{A}$. The imaginary unit “$\:i$” is represented here as $\:i=\sqrt{-1}$. The complex conjugate of $\:\mathcal{A}\left(x,t\right)$ is $\:{\mathcal{A}}^{{\ast}}\left(x,t\right)$. Real-valued constants make up each of the three associated parameters, $\:a,\:b$, and $\:c$. The full non-linearity effects are denoted by the parameter $\:n$; the inter-modal dispersion coefficient is denoted by $\:{a}_{1}$, and the higher-order dispersion and self-steepening coefficients for short pulse terms are represented by $\:{b}_{1}$ and $\:{c}_{1}$, respectively.

## Functioning of modified EM method

In order to solve the QpGI model, it is supposed that Eq. (1) possesses a solution of the following form, resembling a traveling wave solution:

2 $$\:\mathcal{A}\left(x,t\right)=\mathcal{U}\left(\xi\:\right)\:{e}^{i\:\left(\mathcalligra{y}\left(x,t\right)\right)},$$

where

3 $${\mathcalligra{y}}\left( {x,t} \right) = - \kappa ~x + \omega ~t + \theta ~{\text{and}}~\xi = x - \tau ~t.$$

It should be remembered that the phase component of the soliton is represented by $\:\mathcalligra{y}\left(x,t\right)$, and the amplitude function $\:\mathcal{U}\left(\xi\:\right)$ indicates the shape properties of the wave pulse. Furthermore, $\:\kappa\:$, where $\:\theta\:$ is the phase constant, $\:\omega\:$ is the wave number, and $\:\tau\:$ is the soliton velocity, determines the soliton frequency.

By substituting Eq.’s (2) and (3) into Eq. (1), the imaginary part, after integrating with respect to $\:\xi\:,$ will be:

4 $$\left( { - a_{1} - 2a~\kappa - \tau } \right){\mathcal{U}}\left( \xi \right) + \frac{1}{3}c{~\mathcal{U}}\left( \xi \right)^{3} - \frac{{\left( {b_{1} + 2mb_{1} + 2mc_{1} } \right)}}{{1 + 2m}}{\mathcal{U}}\left( \xi \right)^{{1 + 2m}} = 0.$$

After comparing the coefficients of the independent terms, we get the following results:

5 $$\tau = - a_{1} - 2a\kappa ,\,\,c = 0\,\,and\,\,b_{1} = - \frac{4}{5}c_{1}$$

On the other side, the following real component will arise:

6 $$\left( { - a_{1} ~\kappa - a\kappa ^{2} - \omega } \right){\mathcal{U}}\left( \xi \right) - c~\kappa ~{\mathcal{U}}\left( \xi \right)^{3} + b{\mathcal{U}}\left( \xi \right)^{5} - b_{1} \kappa {\mathcal{U}}\left( \xi \right)^{{1 + 2m}} + a{\mathcal{U}^{\prime\prime}}\left( \xi \right) = 0.$$

Now, applying the balance rule to the non-linear ordinary differential equation (Eq. (6)), $\:{\mathcal{U}}^{{\prime\:}{\prime\:}}\left(\xi\:\right)$ and $\:{\mathcal{U}}^{2m+1}\left(\xi\:\right),$ will lead to the balance number $\:n$:

7 $$\:n=\frac{1}{m},\:\:\:\:\:\:\:\:\:m\ge\:2.$$

This is a fractional number, so a transformation function can be assumed in the form:

8 $$\:\mathcal{U}\left({\upxi\:}\right)={\left(\mathcal{G}\left({\upxi\:}\right)\right)}^{\frac{1}{m}}.$$

Letting $\:m=2$ and substituting Eq. (8) with the aid of the obtained parameters in Eq. (5), Eq. (6) gets the form:

9 $$\:{A}_{1}{\mathcal{G}\left({\upxi\:}\right)}^{2}+{A}_{2}{\mathcal{G}\left({\upxi\:}\right)}^{4}-a{{\mathcal{G}}^{{\prime\:}}\left({\upxi\:}\right)}^{2}+2a\mathcal{G}\left({\upxi\:}\right){\mathcal{G}}^{{\prime\:}{\prime\:}}\left({\upxi\:}\right)=0,$$

where

10 $$\:{A}_{1}=4(-{a}_{1}\kappa\:-a\:{\kappa\:}^{2}-\omega\:)\:\:\text{a}\text{n}\text{d}\:{A}_{2}=4\left(b-{b}_{1}\kappa\:\right).$$

Currently, the goal is finding the analytical travelling wave solutions for the QpGI (Eq. (1)) by applying the modified EM technique. In reference^45^, the applied steps are outlined in detail. To express the optical soliton solutions in terms of $\:x$ and $\:t$, we will examine the solutions of Eq. (9). To achieve this, we propose the following form for the solution of Eq. (9):

11 $$\:\mathcal{G}\left({\upxi\:}\right)={\alpha\:}_{0}+\sum\:_{j=1}^{N}\left({\alpha\:}_{j}{\varOmega\:}^{j}\left({\upxi\:}\right)+\frac{{\alpha\:}_{-j}}{{\varOmega\:}^{j}\left({\upxi\:}\right)}\right)+\sum\:_{j=2}^{N}{\stackrel{\sim}{\beta\:}}_{j}{\varOmega\:}^{j-2}\left({\upxi\:}\right){\varOmega\:}^{{\prime\:}}\left({\upxi\:}\right)+\sum\:_{j=-1}^{N}{\beta\:}_{j}{\varOmega\:}^{j}\left({\upxi\:}\right){\varOmega\:}^{{\prime\:}}\left({\upxi\:}\right),$$

where $\:{\alpha\:}_{0}$, $\:{\alpha\:}_{j}$, $\:{\alpha\:}_{-j}$, $\:{\beta\:}_{j}$, and $\:{\stackrel{\sim}{\beta\:}}_{j}$ are constants, to be calculated subsequently in conjunction with $\:\varOmega\:\left({\upxi\:}\right)$ that fulfills the non-linear ODE (Eq. (9)).

Due to the scheme of the modified EM method^45^, $\:\varOmega\:\left({\upxi\:}\right)$ also satisfies

12 $$\Omega ^{\prime}\left( \xi \right) = \in \sqrt {\mathop \sum \limits_{{\begin{array}{*{20}c} {l = 0} \\ {l \ne 5} \\ \end{array} }}^{6} \rangle _{l} \left( {\Omega \left( \xi \right)} \right)^{l} } ,$$

where $\in = \pm 1$. Now, in the supposed solution (Eq. (11)), $\:N\:=\:1$ will be implied by the homogeneous balance for $\:\mathcal{G}\left({\upxi\:}\right){\mathcal{G}}^{{\prime\:}{\prime\:}}\left({\upxi\:}\right)$ in Eq. (9) and any one of $\:{{\mathcal{G}}^{{\prime\:}}\left({\upxi\:}\right)}^{2}$ or $\:{\mathcal{G}\left({\upxi\:}\right)}^{4},$ and thus reaching the formal solution as follows^13,18^:

13 $$\:\mathcal{G}\left({\upxi\:}\right)={\alpha\:}_{0}+{\alpha\:}_{1}\varOmega\:\left({\upxi\:}\right)+\frac{{\alpha\:}_{-1}}{\varOmega\:\left({\upxi\:}\right)}+\frac{{\beta\:}_{1}}{\varOmega\:\left({\upxi\:}\right)}*{\varOmega\:}^{{\prime\:}}\left({\upxi\:}\right).$$

Now, Eq.’s (12) and (13) will be substituted in Eq. (9). By equating to zero all coefficients containing the same order of Ω(ξ), a system of algebraic equations involving the parameters $\:{\alpha\:}_{0},\:{\alpha\:}_{1},\:{\alpha\:}_{-1}$ and $\:{\beta\:}_{1}$ is generated. The resulting algebraic system is solved to obtain these parameter values, which are then inserted into the solution form (Eq. 13) to construct the proposed analytical solutions.

## Solitary wave solutions

### Identification and classification

#### Sets of solutions 1

At $\:{\varrho\:}_{0}={\varrho\:}_{1}={\varrho\:}_{3}={\varrho\:}_{6}=0$, by solving a group of 16 non-linear algebraic equations the resulting sets of solutions will be:

$$\:\text{S}\text{e}\text{t}\:1.1=\left\{{\alpha\:}_{0}={\alpha\:}_{-1}={\beta\:}_{1}=0,\:\:{\varrho\:}_{2}=-\frac{{A}_{1}}{a},\:\:{\alpha\:}_{1}=\in\sqrt{-\frac{3a{\varrho\:}_{4}}{{A}_{2}}}\right\}$$

$$\:\text{S}\text{e}\text{t}\:1.2=\left\{{\alpha\:}_{1}={\beta\:}_{1}={A}_{2}=0,\:\:{\alpha\:}_{0}=\in{\alpha\:}_{-1}\sqrt{\frac{a{\varrho\:}_{4}}{{A}_{1}}},\:\:{\alpha\:}_{-1}\ne\:0,\:\:{\varrho\:}_{2}=-\frac{{A}_{1}}{a}\right\}$$

It is worth mentioning that in Set 1.2, the parameter $\:{\alpha\:}_{0}$ has been represented in terms of the parameter $\:{\alpha\:}_{-1}$. Now, the resulting soliton solutions will be:

14 $${\mathcal{A}}_{1} \left( {x,t} \right) = \sqrt { \in \sqrt { - \frac{{3A_{1} }}{{A_{2} }}} \text{sech} \left( {\left( {x - \tau t} \right)\sqrt { - \frac{{A_{1} }}{a}} } \right)} e^{{i~\left( {y\left( {x,t} \right)} \right)}} ~~~~~,\frac{1}{a}\left( { - a_{1} \kappa - a~\kappa ^{2} - \omega } \right) > 0,$$

15 $${\mathcal{A}}_{2} \left( {x,t} \right) = \sqrt { \in \sqrt { - \frac{{3A_{1} }}{{A_{2} }}} \sec \left( {\left( {x - \tau t} \right)\sqrt {\frac{{A_{1} }}{a}} } \right)} e^{{i~\left( {y\left( {x,t} \right)} \right)}} ~~~~~~,\frac{1}{a}\left( {a_{1} \kappa + a~\kappa ^{2} + \omega } \right) < 0,$$

and

16 $${\mathcal{A}}_{3} \left( {x,t} \right) = \sqrt {\alpha _{{ - 1}} \sqrt {\frac{{a\rangle _{4} }}{{A_{1} }}} \left( { \in + \cosh \left( {\left( {x - \tau t} \right)\sqrt { - \frac{{A_{1} }}{a}} } \right)} \right)} e^{{i~\left( {y\left( {x,t} \right)} \right)}} ~~~,\frac{1}{a}\left( {a_{1} \kappa + a~\kappa ^{2} + \omega } \right) > 0.$$

The solutions obtained in Eq.’s (14–16) are bright, singular periodic and hyperbolic soliton solutions, respectively.

#### Set of solutions 2

At $\:{\varrho\:}_{1}={\varrho\:}_{3}={\varrho\:}_{6}=0,\:\:\:{\varrho\:}_{0}=\frac{{\varrho\:}_{2}^{2}}{4{\varrho\:}_{4}}$, by solving a group of 21 non-linear algebraic equations the resulting sets of solutions will be:

$$\:\text{S}\text{e}\text{t}\:2.1=\left\{{\alpha\:}_{0}={\beta\:}_{1}=0,\:\:{\alpha\:}_{1}=\epsilon\sqrt{\frac{3{A}_{1}{\varrho\:}_{4}}{2{A}_{2}{\varrho\:}_{2}}},\:\:{\alpha\:}_{-1}=\epsilon\sqrt{\frac{3{A}_{1}{\varrho\:}_{4}}{8{A}_{2}{\varrho\:}_{2}}},\:\:a=\frac{{A}_{1}}{2{\varrho\:}_{2}}\right\}$$

$$\:\text{S}\text{e}\text{t}\:2.2=\left\{{\alpha\:}_{0}=0,\:\:{\alpha\:}_{1}=\epsilon\sqrt{\frac{3{A}_{1}{\varrho\:}_{4}}{8{A}_{2}{\varrho\:}_{2}}},\:\:{\alpha\:}_{-1}=\epsilon\sqrt{\frac{3{A}_{1}{\varrho\:}_{2}}{32{A}_{2}{\varrho\:}_{4}}},\:\:{\beta\:}_{1}=\epsilon\sqrt{\frac{3{A}_{1}}{8{A}_{2}{\varrho\:}_{2}}},\:\:a=\frac{{A}_{1}}{2{\varrho\:}_{2}}\right\}$$

$$\:\text{S}\text{e}\text{t}\:2.3=\left\{{\alpha\:}_{0}={\alpha\:}_{1}={\alpha\:}_{-1}=0,\:\:{\beta\:}_{1}=\sqrt{\frac{3{A}_{1}}{2{A}_{2}{\varrho\:}_{2}}},\:\:a=\frac{{A}_{1}}{2{\varrho\:}_{2}}\right\}$$

Now, the resulting solutions will be:

17 $$\begin{aligned} {\mathcal{A}}_{4} \left( {x,t} \right) & = \sqrt {2\smallint \frac{{\rangle _{4} }}{{\rangle _{2} }}\sqrt { - \frac{{3A_{1} }}{{A_{2} }}} \coth \left( {\frac{{\left( {x - \tau t} \right)\sqrt { - \rangle _{2} } }}{{\sqrt 2 }}} \right) + \frac{1}{2}\smallint \sqrt { - \frac{{3A_{1} }}{{A_{2} }}} \tanh \left( {\frac{{\left( {x - \tau t} \right)\sqrt { - \rangle _{2} } }}{{\sqrt 2 }}} \right)} e^{{i~\left( {y\left( {x,t} \right)} \right)}}; \\ & \frac{{a_{1} \kappa + a\kappa ^{2} + \omega }}{{b - b_{1} \kappa }} > 0,~~\rangle _{2} < 0 \\ \end{aligned}$$

18 $$\begin{aligned} {\mathcal{A}}_{5} \left( {x,t} \right) &= \sqrt {\frac{ \in }{4}\sqrt { - \frac{{3A_{1} }}{{A_{2} }}} \left( {2\text{csch} \left( {\frac{{2\left( {x - \tau t} \right)\sqrt { - \rangle _{2} } }}{{\sqrt 2 }}} \right) + \coth \left( {\frac{{\left( {x - \tau t} \right)\sqrt { - \rangle _{2} } }}{{\sqrt 2 }}} \right) + \tanh \left( {\frac{{\left( {x - \tau t} \right)\sqrt { - \rangle _{2} } }}{{\sqrt 2 }}} \right)} \right)} e^{{i~\left( {y\left( {x,t} \right)} \right)}} ; \\ & \frac{{a_{1} \kappa + a\kappa ^{2} + \omega }}{{b - b_{1} \kappa }} > 0,~~\rangle _{2} < 0 \\ \end{aligned}$$

19 $$\begin{aligned} {\mathcal{A}}_{6} \left( {x,t} \right) & = \sqrt { \in \sqrt { - \frac{{3A_{1} }}{{A_{2} }}} \text{csch} \left( {\frac{{2\left( {x - \tau t} \right)\sqrt { - \rangle _{2} } }}{{\sqrt 2 }}} \right)} e^{{i~\left( {y\left( {x,t} \right)} \right)}}; \\ & \frac{{a_{1} \kappa + a\kappa ^{2} + \omega }}{{b - b_{1} \kappa }}\left\langle {0,~~\rangle _{2} } \right\rangle 0, \\ \end{aligned}$$

20 $$\begin{aligned} {\mathcal{A}}_{7} \left( {x,t} \right) & = \sqrt {\frac{1}{2} \in \sqrt {\frac{{3A_{1} }}{{A_{2} }}} \left[ {\frac{{\varrho _{4} }}{{\varrho _{2} }}\cot \left( {\frac{{\left( {x - \tau t} \right)\sqrt {\varrho _{2} } }}{{\sqrt 2 }}} \right) + \tan \left( {\frac{{\left( {x - \tau t} \right)\sqrt {\varrho _{2} } }}{{\sqrt 2 }}} \right)} \right]} e^{{i~\left( {y\left( {x,t} \right)} \right)}}; \\ & \frac{{a_{1} \kappa + a\kappa ^{2} + \omega }}{{b - b_{1} \kappa }}\left\langle {0,~~\varrho _{2} } \right\rangle 0, \\ \end{aligned}$$

21 $$\begin{aligned} {\mathcal{A}}_{8} \left( {x,t} \right) & = \sqrt {\frac{ \in }{4}\sqrt {\frac{{3A_{1} }}{{A_{2} }}} \left[ {2\csc \left( {\frac{{2\left( {x - \tau t} \right)\sqrt {\varrho _{2} } }}{{\sqrt 2 }}} \right) + \cot \left( {\frac{{\left( {x - \tau t} \right)\sqrt {\varrho _{2} } }}{{\sqrt 2 }}} \right) + \tan \left( {\frac{{\left( {x - \tau t} \right)\sqrt {\varrho _{2} } }}{{\sqrt 2 }}} \right)} \right]} e^{{i~\left( {y\left( {x,t} \right)} \right)}}; \\ & \frac{{a_{1} \kappa + a\kappa ^{2} + \omega }}{{b - b_{1} \kappa }}\left\langle {0,~~\varrho _{2} } \right\rangle 0 \\ \end{aligned}$$

and

22 $$\begin{aligned} {\mathcal{A}}_{9} \left( {x,t} \right) = & \sqrt {\frac{1}{2} \in \sqrt {\frac{{3A_{1} }}{{A_{2} }}} \csc \left( {\frac{{2\left( {x - \tau t} \right)\sqrt {\varrho _{2} } }}{{\sqrt 2 }}} \right)} e^{{i~\left( {y\left( {x,t} \right)} \right)}} \\ & ;\frac{{a_{1} \kappa + a\kappa ^{2} + \omega }}{{b - b_{1} \kappa }}\left\langle {0,~~\varrho _{2} } \right\rangle 0. \\ \end{aligned}$$

Equations (17) and (18) yield singular bright combo soliton solutions. Equation (19) represents a singular soliton solution. Similarly, Eqs. (20) and (21) correspond to singular periodic combo soliton solutions, while Eq. (22) describes a singular periodic soliton solution.

#### Set of solutions 3

At $\:{\varrho\:}_{3}={\varrho\:}_{4}={\varrho\:}_{6}=0$, by solving a group of 17 non-linear algebraic equations the resulting sets of solutions will be:

$$\:\text{S}\text{e}\text{t}\:3.1=\left\{{\alpha\:}_{-1}={\beta\:}_{1}={A}_{2}=0,\:\:{\alpha\:}_{1}=-\frac{{A}_{1}{\alpha\:}_{0}}{a{\varrho\:}_{1}},\:\:{\varrho\:}_{2}=-\frac{{A}_{1}}{a}\right\}$$

$$\:\text{S}\text{e}\text{t}\:3.2=\left\{{\alpha\:}_{0}={\alpha\:}_{1}={\beta\:}_{1}=0,\:\:{A}_{1}=-a{\varrho\:}_{2},\:\:{\varrho\:}_{0}=-\frac{{A}_{2}{\alpha\:}_{-1}^{2}}{3a}\right\}$$

$$\:\text{S}\text{e}\text{t}\:3.3=\left\{{{\alpha\:}_{1}={\beta\:}_{1}=0,\:\:\alpha\:}_{0}=\epsilon\sqrt{\frac{a{\varrho\:}_{2}}{{A}_{2}}},\:\:{\alpha\:}_{-1}=\epsilon\sqrt{-\frac{3a{\varrho\:}_{0}}{{A}_{2}}},\:\:{A}_{1}=a{\varrho\:}_{2},\:\:{\varrho\:}_{1}=\epsilon\sqrt{-\frac{4{\varrho\:}_{2}{\varrho\:}_{0}}{3}}\right\}$$

Thus, the obtained solutions will be:

23 $$\:{\mathcal{A}}_{10}\left(x,t\right)=\sqrt{\frac{{\alpha\:}_{0}}{2}\left[1+\text{sinh}\left(2\left(x-\tau\:t\right)\sqrt{-\frac{{A}_{1}}{a}}\right)\right]}{e}^{i\:\left(\mathcalligra{y}\left(x,t\right)\right)}\:\:\:\:\:\:\:\:;{\varrho\:}_{2}>0,\:\:{\varrho\:}_{0}=0,$$

24 $$\:{\mathcal{A}}_{11}\left(x,t\right)=\sqrt{\frac{{\alpha\:}_{0}}{2}\left[1+\text{sin}\left(2\left(x-\tau\:t\right)\sqrt{-\frac{{A}_{1}}{a}}\right)\right]}{e}^{i\:\left(\mathcalligra{y}\left(x,t\right)\right)}\:\:\:\:\:\:\:;{\varrho\:}_{2}<0,\:\:{\varrho\:}_{0}=0,$$

25 $$\:{\mathcal{A}}_{12}\left(x,t\right)=\sqrt{\sqrt{-\frac{3a{\varrho\:}_{2}}{{A}_{2}}}\text{csch}\left(\left(x-\tau\:t\right)\sqrt{{\varrho\:}_{2}}\right)}{e}^{i\:\left(\mathcalligra{y}\left(x,t\right)\right)}\:\:;{\varrho\:}_{0}>0,\:\:{\varrho\:}_{2}>0,\:\:\:{\varrho\:}_{1}=0,$$

and

26 $$\:{\mathcal{A}}_{13}\left(x,t\right)=\sqrt{\sqrt{\frac{3{\text{a}{{\varrho}}}_{2}}{{A}_{2}}}\text{csc}\left(\left(x-\tau\:t\right)\sqrt{-{\varrho\:}_{2}}\right)}{e}^{i\:\left(\mathcalligra{y}\left(x,t\right)\right)}\:;{\varrho\:}_{0}>0,\:\:{\varrho\:}_{2}<0,\:\:\:{\varrho\:}_{1}=0.$$

The solutions derived from Set 3.1, $\:{\mathcal{A}}_{10}\left(x,t\right)$ and $\:{\mathcal{A}}_{11}\left(x,t\right)$ exhibit hyperbolic and periodic soliton characteristics, respectively. Equation (25) yields a singular soliton solution, $\:{\mathcal{A}}_{12}\left(x,t\right)$, while Eq. (26) produces a singular periodic soliton solution, $\:{\mathcal{A}}_{13}\left(x,t\right)$.

#### Set of solutions 4

At $\:{\varrho\:}_{0}={\varrho\:}_{1}={\varrho\:}_{2}={\varrho\:}_{6}=0$, by solving a group of 17 non-linear algebraic equations the resulting sets of solutions will be:

$$\:\text{S}\text{e}\text{t}\:4=\left\{{\alpha\:}_{0}={\alpha\:}_{-1}=0,\:\:{\beta\:}_{1}=\epsilon\sqrt{\frac{-3a}{4{A}_{2}}},\:\:{\varrho\:}_{4}=-\frac{4{A}_{2}{\alpha\:}_{1}^{2}}{3a},\:\:{A}_{1}=0\right\}$$

So, the resulting solutions will be:

27 $$\:{\mathcal{A}}_{14}\left(x,t\right)=\sqrt{\sqrt{-\frac{3a}{{A}_{2}}}\frac{\epsilon{\varrho\:}_{3}(2\sqrt{-{\varrho\:}_{4}}-\left(x-\tau\:t\right){\varrho\:}_{3})}{{\left(x-\tau\:t\right)}^{2}{\varrho\:}_{3}^{2}-4{\varrho\:}_{4}}}{e}^{i\:\left(\mathcalligra{y}\left(x,t\right)\right)};\:\:\:\:\:\:\:\:{\varrho\:}_{4}<0,\:\:\frac{3a}{{A}_{2}}<0,$$

which is a plane wave solution.

#### Set of solutions 5

At $\:{\varrho\:}_{0}={\varrho\:}_{1}={\varrho\:}_{6}=0$, by solving a group of 18 non-linear algebraic equations the resulting set of solutions will be:

$$\:\text{S}\text{e}\text{t}\:5=\left\{{\alpha\:}_{-1}={\beta\:}_{1}=0,\:\:{\alpha\:}_{0}=\epsilon\sqrt{-\frac{{A}_{1}}{{A}_{2}}},\:\:{\alpha\:}_{1}=\epsilon\sqrt{\frac{3a{\varrho\:}_{3}}{16{A}_{1}{A}_{2}}},\:\:{\varrho\:}_{2}=\frac{{A}_{1}}{a},\:\:{\varrho\:}_{4}=\frac{3a{\varrho\:}_{3}^{2}}{16{A}_{1}}\right\}$$

28 $$\:{\mathcal{A}}_{15}\left(x,t\right)=\sqrt{\epsilon\sqrt{-\frac{{A}_{1}}{{A}_{2}}}\left[1-\frac{1}{4}\sqrt{\frac{3}{a{\varrho\:}_{3}}}\left(1+\text{tanh}\left(\frac{1}{2}\left(x-\tau\:t\right)\sqrt{\frac{{A}_{1}}{a}}\right)\right)\right]}{e}^{i\:\left(\mathcalligra{y}\left(x,t\right)\right)},$$

29 $$\:{\mathcal{A}}_{16}\left(x,t\right)=\sqrt{\epsilon\sqrt{-\frac{{A}_{1}}{{A}_{2}}}\left[1+\sqrt{\frac{-3}{16a{\varrho\:}_{3}}}\left(1+\text{coth}\left(\frac{1}{2}\left(x-\tau\:t\right)\sqrt{\frac{{A}_{1}}{a}}\right)\right)\right]}{e}^{i\:\left(\mathcalligra{y}\left(x,t\right)\right)},$$

and

30 $$\:{\mathcal{A}}_{17}\left(x,t\right)=\sqrt{\epsilon\sqrt{-\frac{{A}_{1}}{{A}_{2}}}\left[1+\frac{\epsilon\:\sqrt{-3a{\varrho\:}_{3}}\text{sech}\left(\frac{1}{2}\left(x-\tau\:t\right)\sqrt{\frac{{A}_{1}}{a}}\right)}{4\left(-{A}_{1}+\frac{1}{2}a\sqrt{3{\varrho\:}_{3}^{2}}\text{tanh}\left(\frac{1}{2}\left(x-\tau\:t\right)\sqrt{\frac{{A}_{1}}{a}}\right)\right)}\right]}{e}^{i\:\left(\mathcalligra{y}\left(x,t\right)\right)}.$$

The solutions obtained in Eq.’s (28–30) are dark, singular and shock soliton solutions.

#### Set of solutions 6

When $\:{\varrho\:}_{2}={\varrho\:}_{4}={\varrho\:}_{6}=0,\:{\varrho\:}_{3}>0$, by solving a group of 19 non-linear algebraic equations, the resulting set of solutions will be:

$$\:\text{S}\text{e}\text{t}\:6=\left\{{\alpha\:}_{0}=\epsilon\sqrt{\frac{{A}_{1}}{2{A}_{2}}},\:\:{\alpha\:}_{1}={\beta\:}_{1}=0,\:\:{\alpha\:}_{-1}=-\frac{\epsilon}{2a{\varrho\:}_{3}}\sqrt{\frac{{A}_{1}^{3}}{2{A}_{2}}},\:\:{\varrho\:}_{0}=\frac{{A}_{1}^{3}}{24{a}^{3}{\varrho\:}_{3}^{2}},\:\:{\varrho\:}_{1}=-\frac{{A}_{1}^{2}}{3{a}^{2}{\varrho\:}_{3}}\right\}$$

The results obtained by set 6 are:

31 $$\:{\mathcal{A}}_{18}\left(x,t\right)=\sqrt{\epsilon\sqrt{\frac{{A}_{1}}{2{A}_{2}}}\left(1-\frac{{A}_{1}}{2a{\varrho\:}_{3}}\frac{1}{\wp\left(\frac{1}{2}\sqrt{{\varrho\:}_{3}}\left(x-\tau\:t\right);-\frac{4{\varrho\:}_{1}}{{\varrho\:}_{3}},-\frac{4{\varrho\:}_{0}}{{\varrho\:}_{3}}\right)}\right)}{e}^{i\:\left(\mathcalligra{y}\left(x,t\right)\right)},$$

where ℘$\left(\frac{1}{2}\sqrt{{\varrho\:}_{3}}\left(x-\tau\:t\right);-\frac{4{\varrho\:}_{1}}{{\varrho\:}_{3}},-\frac{4{\varrho\:}_{0}}{{\varrho\:}_{3}}\right)$ is the Weierstrass ℘-function.

#### Set of solutions 7

When $\:{\varrho\:}_{1}={\varrho\:}_{3}={\varrho\:}_{6}=0$, the resulting set of the solutions for the algebraic set of equations will be:

$$\:\text{S}\text{e}\text{t}\:7.1=\left\{{\alpha\:}_{0}={\beta\:}_{1}=0,{\alpha\:}_{-1}=\epsilon\sqrt{\frac{3{\text{a}{{\varrho}}}_{0}}{{A}_{2}}},{\alpha\:}_{1}=\frac{\epsilon{A}_{1}}{4}\sqrt{\frac{3}{a{A}_{2}{\varrho\:}_{0}}},{\varrho\:}_{2}=\frac{{A}_{1}}{2a},{\varrho\:}_{4}=\frac{{A}_{1}^{2}}{16{a}^{2}{\varrho\:}_{0}}\right\}$$

$$\:\text{S}\text{e}\text{t}\:7.2=\left\{{\alpha\:}_{0}=0,{\alpha\:}_{-1}=\frac{\epsilon}{2}\sqrt{\frac{3{\text{a}{{\varrho}}}_{0}}{{A}_{2}}},{\beta\:}_{1}=\frac{\epsilon}{2}\sqrt{\frac{3a}{{A}_{2}}},{\alpha\:}_{1}=\frac{\epsilon{A}_{1}}{8}\sqrt{\frac{3}{{\text{a}\text{A}}_{2}{\varrho\:}_{0}}},{\varrho\:}_{2}=\frac{{A}_{1}}{2a},{\varrho\:}_{4}=\frac{{A}_{1}^{2}}{16{a}^{2}{\varrho\:}_{0}}\right\}$$

$$\:\text{S}\text{e}\text{t}\:7.3=\left\{{\alpha\:}_{0}={\alpha\:}_{1}={\alpha\:}_{-1}=0,{\beta\:}_{1}=\sqrt{\frac{3a}{{A}_{2}}},{\varrho\:}_{2}=\frac{{A}_{1}}{2a},{\varrho\:}_{4}=\frac{{A}_{1}^{2}}{16{a}^{2}{\varrho\:}_{0}}\right\}$$

The solutions presented in Eqs. (32–48) are expressed in terms of Jacobi elliptic functions $\:dn\left(\xi\:,\mu\:\right),\:sn\left(\xi\:,\mu\:\right),\:nc\left(\xi\:,\mu\:\right),\:sc\left(\xi\:,\mu\:\right),\:nd\left(\xi\:,\mu\:\right),\:cd\left(\xi\:,\mu\:\right),\:sd\left(\xi\:,\mu\:\right),\:cn\left(\xi\:,\mu\:\right)\:$and $\:dn\left(\xi\:,\mu\:\right)$. It is observed that variations in the parameters $\:{\varrho\:}_{0},\:{\varrho\:}_{2}$ and $\:{\varrho\:}_{4}$ result in change of the solution forms.

At $\:\left\{{\varrho\:}_{0}=1,\:{\varrho\:}_{2}=-\left(1+{\mu\:}^{2}\right),{\varrho\:}_{4}={\mu\:}^{2}\:\right\}$, the solutions will be:

32 $$\:{\mathcal{A}}_{19}\left(x,t\right)=\sqrt{\frac{\epsilon}{4\:cd\left(x-\tau\:t,\mu\:\right)}\left[{A}_{1}\sqrt{\frac{3}{a{A}_{2}{\varrho\:}_{0}}}{cd}^{2}\left(x-\tau\:t,\mu\:\right)+4\sqrt{\frac{3a{\varrho\:}_{0}}{{A}_{2}}}\right]}{e}^{i\:\left(\mathcalligra{y}\left(x,t\right)\right)},$$

33 $$\:{\mathcal{A}}_{20}\left(x,t\right)=\sqrt{\frac{\epsilon}{8\:cd\left(x-\tau\:t,\mu\:\right)}\left[4\sqrt{\frac{3a{\varrho\:}_{0}}{{A}_{2}}}+4\left(\mu\:-1\right)nd\left(x-\tau\:t,\mu\:\right)\:\text{s}d\left(x-\tau\:t,\mu\:\right)\sqrt{\frac{3a}{{A}_{2}}}+{cd}^{2}\left(x-\tau\:t,\mu\:\right){A}_{1}\sqrt{\frac{3}{{\text{a}\text{A}}_{2}{\varrho\:}_{0}}}\right]}{e}^{i\:\left(\mathcalligra{y}\left(x,t\right)\right)},$$

and

34 $$\:{\mathcal{A}}_{21}\left(x,t\right)=\sqrt{(\mu\:-1)\sqrt{\frac{3a}{{A}_{2}}}\frac{nd\left(x-\tau\:t,\mu\:\right)\:\text{s}d\left(x-\tau\:t,\mu\:\right)}{cd\left(x-\tau\:t,\mu\:\right)}}{e}^{i\:\left(\mathcalligra{y}\left(x,t\right)\right)}.$$

At $\:\left\{{\varrho\:}_{0}={\mu\:}^{2}-1,\:{\varrho\:}_{2}=2-{\mu\:}^{2},{\varrho\:}_{4}=-1\:\right\}$, the solutions will be:

35 $$\:{\mathcal{A}}_{22}\left(x,t\right)=\sqrt{-\frac{{dn}^{2}\left(x-\tau\:t,\mu\:\right){A}_{1}\sqrt{\frac{3}{a{A}_{2}{\varrho\:}_{0}}}+4\sqrt{\frac{3a{\varrho\:}_{0}}{{A}_{2}}}}{4\:dn\left(x-\tau\:t,\mu\:\right)}}{e}^{i\:\left(\mathcalligra{y}\left(x,t\right)\right)},$$

36 $$\:{\mathcal{A}}_{23}\left(x,t\right)=\sqrt{\frac{4\mu\:\:cn\left(x-\tau\:t,\mu\:\right)\:sn\left(x-\tau\:t,\mu\:\right)\sqrt{\frac{3a}{{A}_{2}}}-4\sqrt{\frac{3a{\varrho\:}_{0}}{{A}_{2}}}-{A}_{1}\sqrt{\frac{3}{{\text{a}\text{A}}_{2}{\varrho\:}_{0}}}{dn}^{2}\left(x-\tau\:t,\mu\:\right)}{8\:dn\left(x-\tau\:t,\mu\:\right)}}{e}^{i\:\left(\mathcalligra{y}\left(x,t\right)\right)},$$

and

37 $$\:{\mathcal{A}}_{24}\left(x,t\right)=\sqrt{\frac{\epsilon\left({A}_{1}\sqrt{\frac{3}{a{A}_{2}{\varrho\:}_{0}}}{nc}^{2}\left(x-\tau\:t,\mu\:\right)+4\sqrt{\frac{3a{\varrho\:}_{0}}{{A}_{2}}}\right)}{4\:nc\left(x-\tau\:t,\mu\:\right)}}{e}^{i\:\left(\mathcalligra{y}\left(x,t\right)\right)}.$$

At $\:\left\{{\varrho\:}_{0}=-{\mu\:}^{2},\:{\varrho\:}_{2}=2{\mu\:}^{2}-1,{\varrho\:}_{4}=-{\mu\:}^{2}+1\:\right\}$, the solutions will be

38 $$\:{\mathcal{A}}_{25}\left(x,t\right)=\sqrt{\frac{\epsilon\left(4\:dc\left(x-\tau\:t,\mu\:\right)\:sc\left(x-\tau\:t,\mu\:\right)\sqrt{\frac{3a}{{A}_{2}}}+{A}_{1}\sqrt{\frac{3}{{\text{a}\text{A}}_{2}{\varrho\:}_{0}}}{nc}^{2}\left(x-\tau\:t,\mu\:\right)+4\sqrt{\frac{3a{\varrho\:}_{0}}{{A}_{2}}}\right)}{8\:nc\left(x-\tau\:t,\mu\:\right)}}{e}^{i\:\left(\mathcalligra{y}\left(x,t\right)\right)},$$

and

39 $$\:{\mathcal{A}}_{26}\left(x,t\right)=\sqrt{\sqrt{\frac{3a}{{A}_{2}}}\frac{dc\left(x-\tau\:t,\mu\:\right)\:sc\left(x-\tau\:t,\mu\:\right)\:}{nc\left(x-\tau\:t,\mu\:\right)}}{e}^{i\:\left(\mathcalligra{y}\left(x,t\right)\right)}.$$

At $\:\left\{{\varrho\:}_{0}=-1,\:{\varrho\:}_{2}=-{\mu\:}^{2}+2,{\varrho\:}_{4}={\mu\:}^{2}-1\right\}$, the solutions will be

40 $$\:{\mathcal{A}}_{27}\left(x,t\right)=\sqrt{\frac{\epsilon\left({nd}^{2}\left(x-\tau\:t,\mu\:\right){A}_{1}\sqrt{\frac{3}{a{A}_{2}{\varrho\:}_{0}}}+4\sqrt{\frac{3a{\varrho\:}_{0}}{{A}_{2}}}\right)}{4\:nd\left(x-\tau\:t,\mu\:\right)}}{e}^{i\:\left(\mathcalligra{y}\left(x,t\right)\right)},$$

41 $$\:{\mathcal{A}}_{28}\left(x,t\right)=\sqrt{\frac{\epsilon\left(4\:\mu\:\:cd\left(x-\tau\:t,\mu\:\right)\:sd\left(x-\tau\:t,\mu\:\right)\sqrt{\frac{3a}{{A}_{2}}}+{nd}^{2}\left(x-\tau\:t,\mu\:\right){A}_{1}\sqrt{\frac{3}{{\text{a}\text{A}}_{2}{\varrho\:}_{0}}}+4\sqrt{\frac{3a\:{\varrho\:}_{0}}{{A}_{2}}}\right)}{8\:nd\left(x-\tau\:t,\mu\:\right)}}{e}^{i\:\left(\mathcalligra{y}\left(x,t\right)\right)}$$

and

42 $$\:{\mathcal{A}}_{29}\left(x,t\right)=\sqrt{\mu\:\sqrt{\frac{3a}{{A}_{2}}}\frac{cd\left(x-\tau\:t,\mu\:\right)\:sd\left(x-\tau\:t,\mu\:\right)}{nd\left(x-\tau\:t,\mu\:\right)}}{e}^{i\:\left(\mathcalligra{y}\left(x,t\right)\right)}.$$

At $\:\left\{{\varrho\:}_{0}={\mu\:}^{2}-2{\mu\:}^{3}+{\mu\:}^{4},\:{\varrho\:}_{2}=-\frac{4}{\mu\:},{\varrho\:}_{4}=-1+6\mu\:+{\mu\:}^{2}\right\}$, the obtained solutions will be:

43 $$\:{\mathcal{A}}_{30}\left(x,t\right)=\sqrt{\epsilon\:\mu\:\:{A}_{1}\sqrt{\frac{3}{a{A}_{2}{\varrho\:}_{0}}}\frac{cn\left(x-\tau\:t,\mu\:\right)\:dn\left(x-\tau\:t,\mu\:\right)}{4\left(1+\mu\:\:sn\left(x-\tau\:t,\mu\:\right)\right)}+\frac{\epsilon}{\mu\:}\sqrt{\frac{3a{\varrho\:}_{0}}{{A}_{2}}}\frac{\left(1+\mu\:\:sn\left(x-\tau\:t,\mu\:\right)\right)}{cn\left(x-\tau\:t,\mu\:\right)\:dn\left(x-\tau\:t,\mu\:\right)}}{e}^{i\:\left(\mathcalligra{y}\left(x,t\right)\right)},$$

44 $$\:{\mathcal{A}}_{31}\left(x,t\right)=\sqrt{\frac{\epsilon}{2}\sum\:_{i=1}^{9}{\mathcalligra{s}}_{\left(i,1\right)}\left(\mu\:\right){\mathcalligra{k}}_{\left(i,1\right)}\left(\xi\:,\mu\:\right)}{e}^{i\:\left(\mathcalligra{y}\left(x,t\right)\right)},$$

where

$$\:{\mathcalligra{s}}_{\left(\text{1,1}\right)}\left(\mu\:\right)=-\mu\:\sqrt{\frac{3a}{{A}_{2}}};$$ $$\:{\mathcalligra{k}}_{\left(\text{1,1}\right)}\left(\xi\:,\mu\:\right)=\frac{cn\left(x-\tau\:t,\mu\:\right)\:dn\left(x-\tau\:t,\mu\:\right)\:}{{(1+\mu\:\:sn\left(x-\tau\:t,\mu\:\right))}^{2}}$$

$$\:{\mathcalligra{s}}_{\left(\text{1,2}\right)}\left(\mu\:\right)=-{\mu\:}^{2}\sqrt{\frac{3a}{{A}_{2}}};$$ $$\:{\mathcalligra{k}}_{\left(\text{1,2}\right)}\left(\xi\:,\mu\:\right)=\frac{cn\left(x-\tau\:t,\mu\:\right)\:dn\left(x-\tau\:t,\mu\:\right)\:sn\left(x-\tau\:t,\mu\:\right)}{{\left[1+\mu\:\:sn\left(x-\tau\:t,\mu\:\right)\right]}^{2}}$$

$$\:{\mathcalligra{s}}_{\left(\text{1,3}\right)}\left(\mu\:\right)=-\mu\:\sqrt{\frac{3a}{{A}_{2}}};$$ $$\:{\mathcalligra{k}}_{\left(\text{1,3}\right)}\left(\xi\:,\mu\:\right)=\frac{cn\left(x-\tau\:t,\mu\:\right)\:sn\left(x-\tau\:t,\mu\:\right)}{dn\left(x-\tau\:t,\mu\:\right)\left[1+\mu\:\:sn\left(x-\tau\:t,\mu\:\right)\right]}$$

$$\:{\mathcalligra{s}}_{\left(\text{1,4}\right)}\left(\mu\:\right)=-\sqrt{\frac{3a}{{A}_{2}}};$$ $$\:{\mathcalligra{k}}_{\left(\text{1,4}\right)}\left(\xi\:,\mu\:\right)=\frac{dn\left(x-\tau\:t,\mu\:\right)\:sn\left(x-\tau\:t,\mu\:\right)}{cn\left(x-\tau\:t,\mu\:\right)\:\left[1+\mu\:\:\:sn\left(x-\tau\:t,\mu\:\right)\right]}$$

$$\:{\mathcalligra{s}}_{\left(\text{1,5}\right)}\left(\mu\:\right)=-{\mu\:}^{2}\sqrt{\frac{3a}{{A}_{2}}};$$ $$\:{\mathcalligra{k}}_{\left(\text{1,5}\right)}\left(\xi\:,\mu\:\right)=\frac{cn\left(x-\tau\:t,\mu\:\right)\:{sn}^{2}\left(x-\tau\:t,\mu\:\right)\:}{dn\left(x-\tau\:t,\mu\:\right)\:\left[1+\mu\:\:sn\left(x-\tau\:t,\mu\:\right)\right]}$$

$$\:{\mathcalligra{s}}_{\left(\text{1,6}\right)}\left(\mu\:\right)=-\mu\:\sqrt{\frac{3a}{{A}_{2}}};$$ $$\:{\mathcalligra{k}}_{\left(\text{1,6}\right)}\left(\xi\:,\mu\:\right)=\frac{dn\left(x-\tau\:t,\mu\:\right)\:{sn}^{2}\left(x-\tau\:t,\mu\:\right)}{cn\left(x-\tau\:t,\mu\:\right)\:\left[1+\mu\:\:sn\left(x-\tau\:t,\mu\:\right)\right]}$$

$$\:{\mathcalligra{s}}_{\left(\text{1,7}\right)}\left(\mu\:\right)=\frac{1}{\mu\:}\sqrt{\frac{{3\text{a}{{\varrho}}}_{0}}{{A}_{2}}};$$ $$\:{\mathcalligra{k}}_{\left(\text{1,7}\right)}\left(\xi\:,\mu\:\right)=\frac{1}{cn\left(x-\tau\:t,\mu\:\right)\:dn\left(x-\tau\:t,\mu\:\right)}$$

$$\:{\mathcalligra{s}}_{\left(\text{1,8}\right)}\left(\mu\:\right)=\sqrt{\frac{{3\text{a}{{\varrho}}}_{0}}{{A}_{2}}};$$ $$\:{\mathcalligra{k}}_{\left(\text{1,8}\right)}\left(\xi\:,\mu\:\right)=\frac{sn\left(x-\tau\:t,\mu\:\right)}{cn\left(x-\tau\:t,\mu\:\right)\:dn\left(x-\tau\:t,\mu\:\right)}$$

$$\:{\mathcalligra{s}}_{\left(\text{1,9}\right)}\left(\mu\:\right)=\frac{\mu\:{A}_{1}}{4}\sqrt{\frac{3}{{\text{a}\text{A}}_{2}{\varrho\:}_{0}}};$$$$\:{\mathcalligra{k}}_{\left(\text{1,9}\right)}\left(\xi\:,\mu\:\right)=\frac{cn\left(x-\tau\:t,\mu\:\right)\:dn\left(x-\tau\:t,\mu\:\right)}{1+\mu\:\:\:sn\left(x-\tau\:t,\mu\:\right)}$$and

45 $$\:{\mathcal{A}}_{32}\left(x,t\right)=\sqrt{\sum\:_{i=1}^{6}{\mathcalligra{s}}_{\left(i,1\right)}\left(\mu\:\right){\mathcalligra{k}}_{\left(i,1\right)}\left(\xi\:,\mu\:\right)}{e}^{i\:\left(\mathcalligra{y}\left(x,t\right)\right)}.$$

At $$\:\left\{{\varrho\:}_{0}=\frac{1}{4},\:{\varrho\:}_{2}=\frac{1}{2}{\mu\:}^{2}-1,{\varrho\:}_{4}=\frac{{\mu\:}^{4}}{4}\right\}$$

46 $$\:{\mathcal{A}}_{33}\left(x,t\right)=\sqrt{\frac{\epsilon\:{A}_{1}}{4}\sqrt{\frac{3}{a{A}_{2}{\varrho\:}_{0}}}\frac{sn\left(x-\tau\:t,\mu\:\right)}{1+\mu\:\:dn\left(x-\tau\:t,\mu\:\right)\:}+\epsilon\sqrt{\frac{3a{\varrho\:}_{0}}{{A}_{2}}}\frac{1+\mu\:\:dn\left(x-\tau\:t,\mu\:\right)}{sn\left(x-\tau\:t,\mu\:\right)}}{e}^{i\:\left(\mathcalligra{y}\left(x,t\right)\right)},$$

47 $$\:{\mathcal{A}}_{34}\left(x,t\right)=\sqrt{\frac{\epsilon}{2}\sum\:_{i=1}^{7}{\mathcalligra{s}}_{\left(2,i\right)}\left(\mu\:\right){\mathcalligra{k}}_{\left(2,i\right)}\left(\xi\:,\mu\:\right)}{e}^{i\:\left(\mathcalligra{y}\left(x,t\right)\right)},$$

where

$$\:{\mathcalligra{s}}_{\left(\text{2,1}\right)}\left(\mu\:\right)=\sqrt{\frac{3a}{{A}_{2}}};$$ $$\:{\mathcalligra{k}}_{\left(\text{2,1}\right)}\left(\xi\:,\mu\:\right)=\frac{cn\left(x-\tau\:t,\mu\:\right)\:dn\left(x-\tau\:t,\mu\:\right)}{\left[1+\mu\:\:dn\left(x-\tau\:t,\mu\:\right)\right]\:sn\left(x-\tau\:t,\mu\:\right)}$$

$$\:{\mathcalligra{s}}_{\left(\text{2,2}\right)}\left(\mu\:\right)=\mu\:\sqrt{\frac{3a}{{A}_{2}}};$$ $$\:{\mathcalligra{k}}_{\left(\text{2,2}\right)}\left(\xi\:,\mu\:\right)=\frac{cn\left(x-\tau\:t,\mu\:\right)\:{dn}^{2}\left(x-\tau\:t,\mu\:\right)}{\left[1+\mu\:\:dn\left(x-\tau\:t,\mu\:\right)\right]\:\:sn\left(x-\tau\:t,\mu\:\right)}$$

$$\:{\mathcalligra{s}}_{\left(\text{2,3}\right)}\left(\mu\:\right)={\mu\:}^{2}\sqrt{\frac{3a}{{A}_{2}}};$$ $$\:{\mathcalligra{k}}_{\left(\text{2,3}\right)}\left(\xi\:,\mu\:\right)=\frac{cn\left(x-\tau\:t,\mu\:\right)\:sn\left(x-\tau\:t,\mu\:\right)}{{(1+\mu\:\:dn\left(x-\tau\:t,\mu\:\right))}^{2}}$$

$$\:{\mathcalligra{s}}_{\left(\text{2,4}\right)}\left(\mu\:\right)={\mu\:}^{3}\sqrt{\frac{3a}{{A}_{2}}};$$ $$\:{\mathcalligra{k}}_{\left(\text{2,4}\right)}\left(\xi\:,\mu\:\right)=\frac{cn\left(x-\tau\:t,\mu\:\right)\:\:dn\left(x-\tau\:t,\mu\:\right)\:sn\left(x-\tau\:t,\mu\:\right)}{{(1+\mu\:\:dn\left(x-\tau\:t,\mu\:\right))}^{2}}$$

$$\:{\mathcalligra{s}}_{\left(\text{2,5}\right)}\left(\mu\:\right)=\sqrt{\frac{{3\text{a}{{\varrho}}}_{0}}{{A}_{2}}};$$ $$\:{\mathcalligra{k}}_{\left(\text{2,5}\right)}\left(\xi\:,\mu\:\right)=\frac{1}{sn\left(x-\tau\:t,\mu\:\right)}$$

$$\:{\mathcalligra{s}}_{\left(\text{2,6}\right)}\left(\mu\:\right)=\mu\:\sqrt{\frac{{3\text{a}{{\varrho}}}_{0}}{{A}_{2}}};$$ $$\:{\mathcalligra{k}}_{\left(\text{2,6}\right)}\left(\xi\:,\mu\:\right)=\frac{dn\left(x-\tau\:t,\mu\:\right)}{sn\left(x-\tau\:t,\mu\:\right)}$$

$$\:{\mathcalligra{s}}_{\left(\text{2,7}\right)}\left(\mu\:\right)=\frac{{A}_{1}}{4}\sqrt{\frac{31}{{\text{a}\text{A}}_{2}{\varrho\:}_{0}}};$$$$\:{\mathcalligra{k}}_{\left(\text{2,7}\right)}\left(\xi\:,\mu\:\right)=\frac{sn\left(x-\tau\:t,\mu\:\right)}{1+\mu\:\:dn\left(x-\tau\:t,\mu\:\right)}$$and

48 $$\:{\mathcal{A}}_{35}\left(x,t\right)=\sqrt{\frac{\epsilon}{2}\sum\:_{i=1}^{4}{\mathcalligra{s}}_{\left(i,2\right)}\left(\mu\:\right){\mathcalligra{k}}_{\left(i,2\right)}\left(\xi\:,\mu\:\right)}{e}^{i\:\left(\mathcalligra{y}\left(x,t\right)\right)}.$$

### Graphical representation of some obtained solutions

In addition to shock and plane wave solutions, the QpGI model includes a wide range of another traveling wave soliton solutions, such as bright, singular periodic, singular bright combo, dark, singular, Jacobi elliptic and hyperbolic soliton solutions. To fully understand the QpGI model, these solutions must be analyzed through various graphs. This section will visualize the physical behavior of these solutions in both 2D and 3D representations. Due to the component $\:{e}^{i\:\left(\mathcalligra{y}\left(x,t\right)\right)}$ in Eq. (2), the derived solutions are complex.

**Fig. 1:**
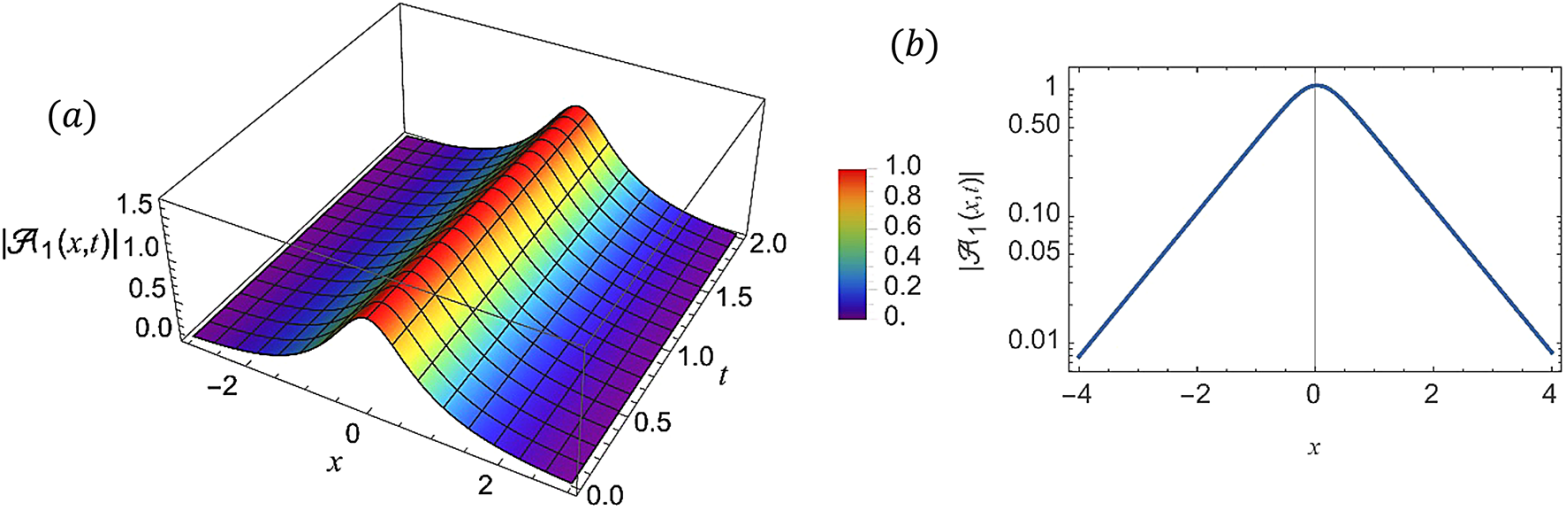
(**a**) 3D graph for $\:\left|{\mathcal{A}}_{1}\left(x,t\right)\right|$ showing the bright soliton solution’s physical behavior as a function of x and t variables. (Eq. (14)). (**b**) 2D graph for $\:\left|{\mathcal{A}}_{1}\left(x,t\right)\right|$ as a function of x at t=0.3.

**Fig. 2:**
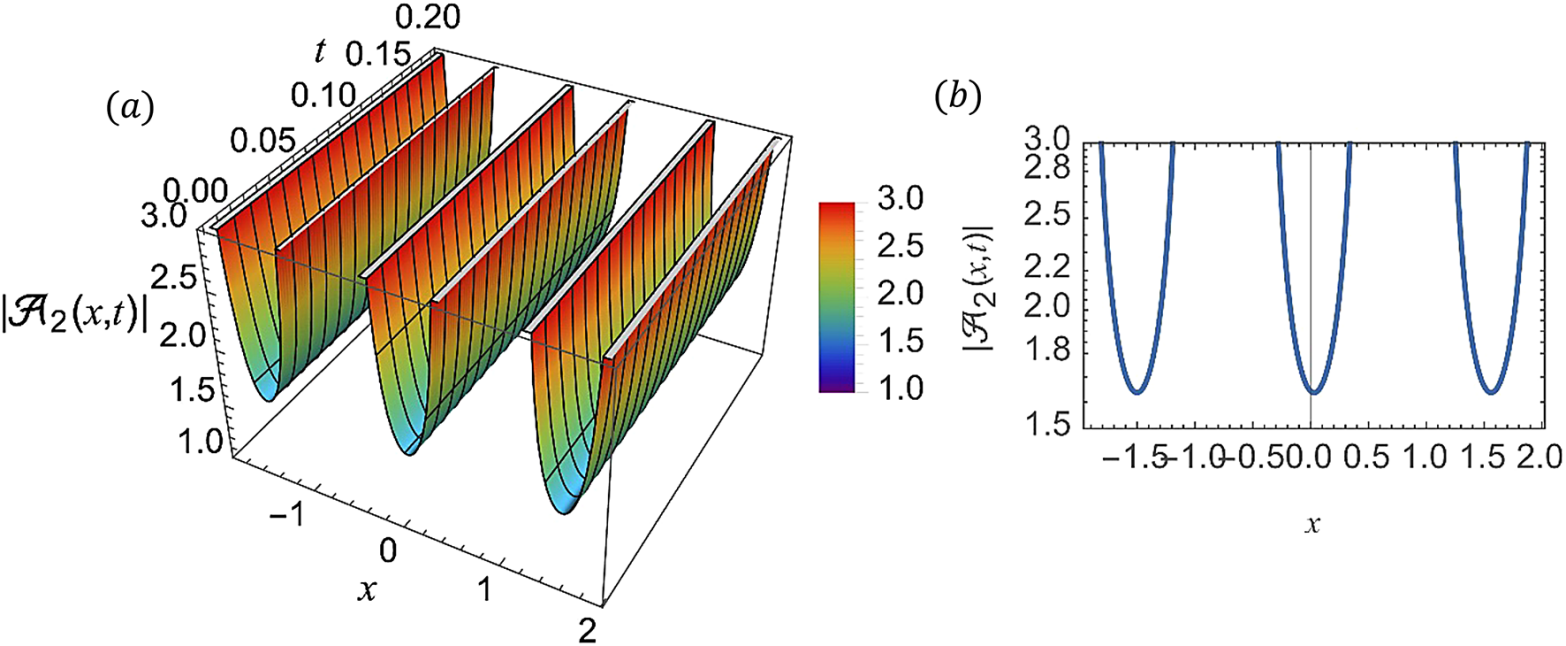
(**a**) 3D graph for $\:\left|{\mathcal{A}}_{2}\left(x,t\right)\right|$ showing the singular periodic soliton solution’s physical behavior as a function of * x* and* t* variables. (Eq. (15)). (**b**) 2D graph for $\:\left|{\mathcal{A}}_{2}\left(x,t\right)\right|$ as a function of * x* at* t=0.3*

**Fig. 3:**
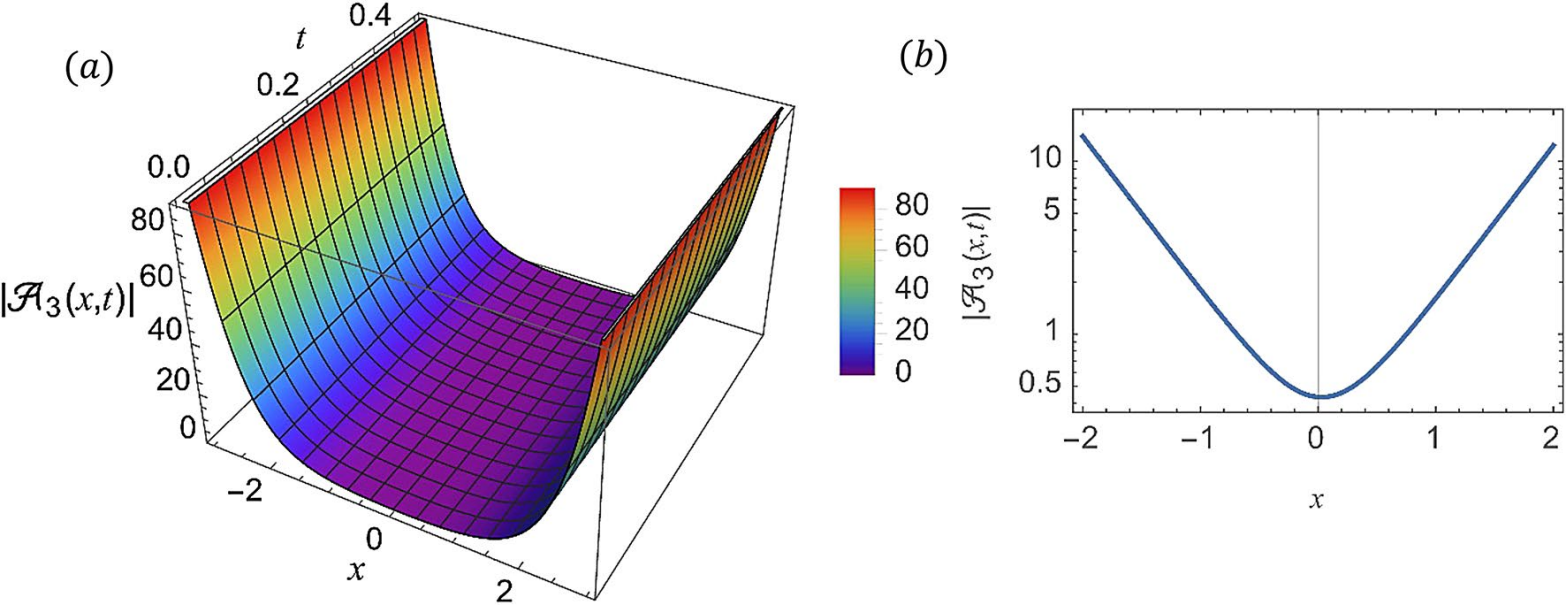
(**a**) 3D graph for $\:\left|{\mathcal{A}}_{3}\left(x,t\right)\right|$ showing the hyperbolic soliton solution’s physical behavior as a function of $\:x$ and $\:t$ variables. (Eq. (16)). (**b**) 2D graph for $\:\left|{\mathcal{A}}_{3}\left(x,t\right)\right|$ as a function of $\:x$ at $\:t=0.3$.

**Fig. 4:**
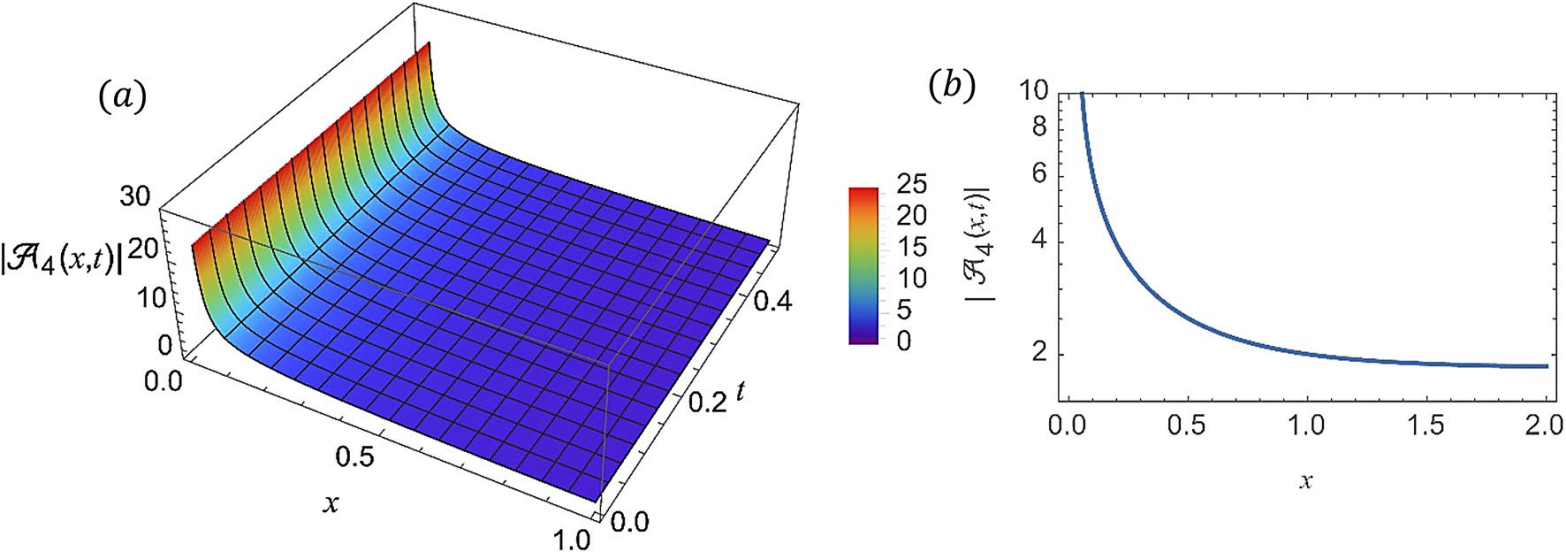
(**a**) 3D graph for $\:\left|{\mathcal{A}}_{4}\left(x,t\right)\right|$ showing the singular bright combo soliton solution’s physical behavior as a function of * x* and * t* variables. (Eq. (17)). (**b**) 2D graph for $\:\left|{\mathcal{A}}_{3}\left(x,t\right)\right|$ as a function of * x* at $\:t=0.3$.

**Fig. 5:**
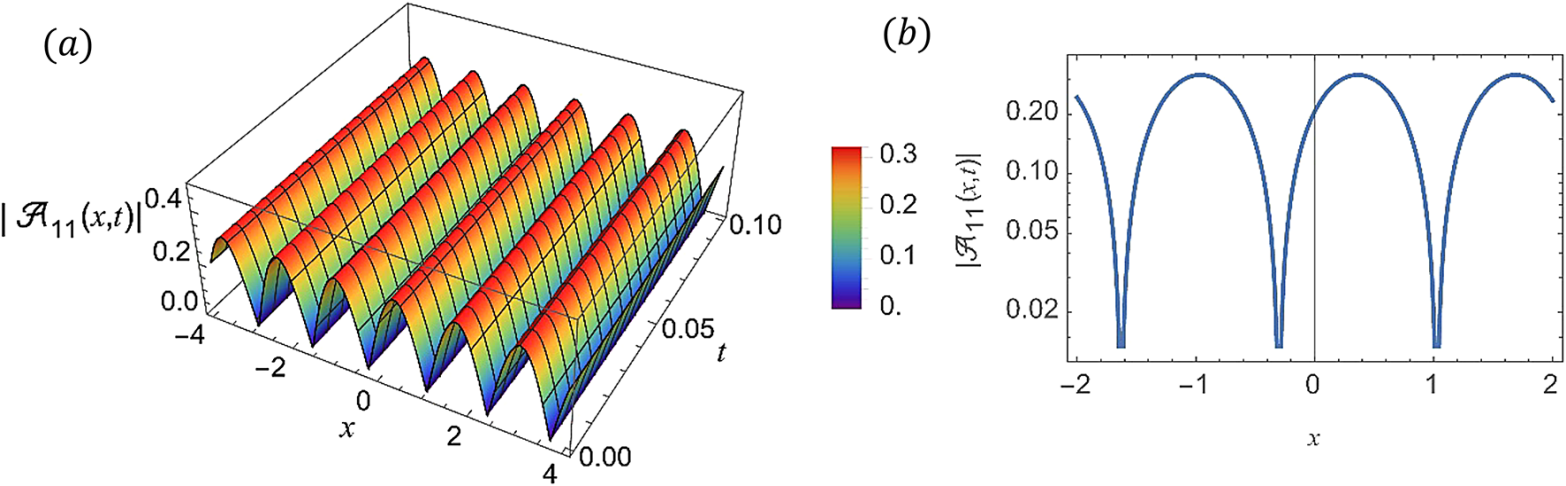
(**a**) 3D graph for $\:\left|{\mathcal{A}}_{11}\left(x,t\right)\right|$ showing the periodic soliton solution’s physical behavior as a function of * x* and * t* variables. (Eq. (24)). (**b**) 2D graph for $\:\left|{\mathcal{A}}_{11}\left(x,t\right)\right|$ as a function of * x* at $\:t=0.3$.

**Fig. 6:**
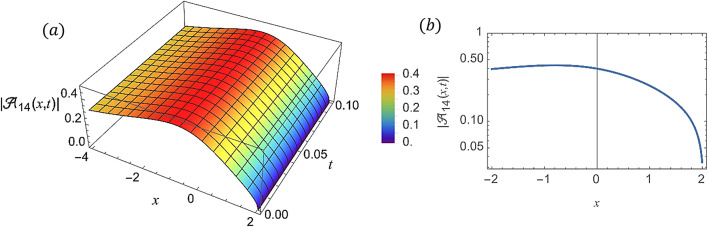
(**a**) 3D graph for $\:\left|{\mathcal{A}}_{14}\left(x,t\right)\right|$ showing the plane wave solution’s physical behavior as a function of * x* and* t* variables. (Eq. (27)). (**b**) 2D graph for $\:\left|{\mathcal{A}}_{14}\left(x,t\right)\right|$ as a function of * t* at $\:t=0.3$.

**Fig. 7:**
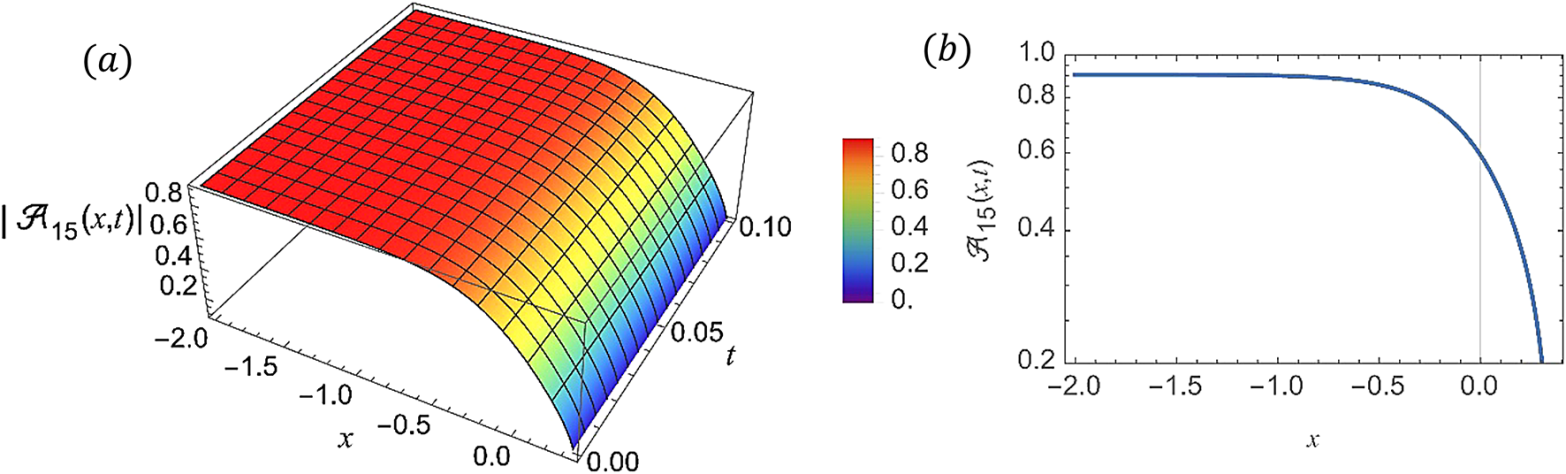
(**a**) 3D graph for $\:\left|{\mathcal{A}}_{15}\left(x,t\right)\right|$ showing the dark soliton solution’s physical behavior as a function of* x* and* t* variables. (Eq. (28)). (**b**) 2D graph for $\:\left|{\mathcal{A}}_{15}\left(x,t\right)\right|$ as a function of* x* at $\:t=0.3$.

**Fig. 8:**
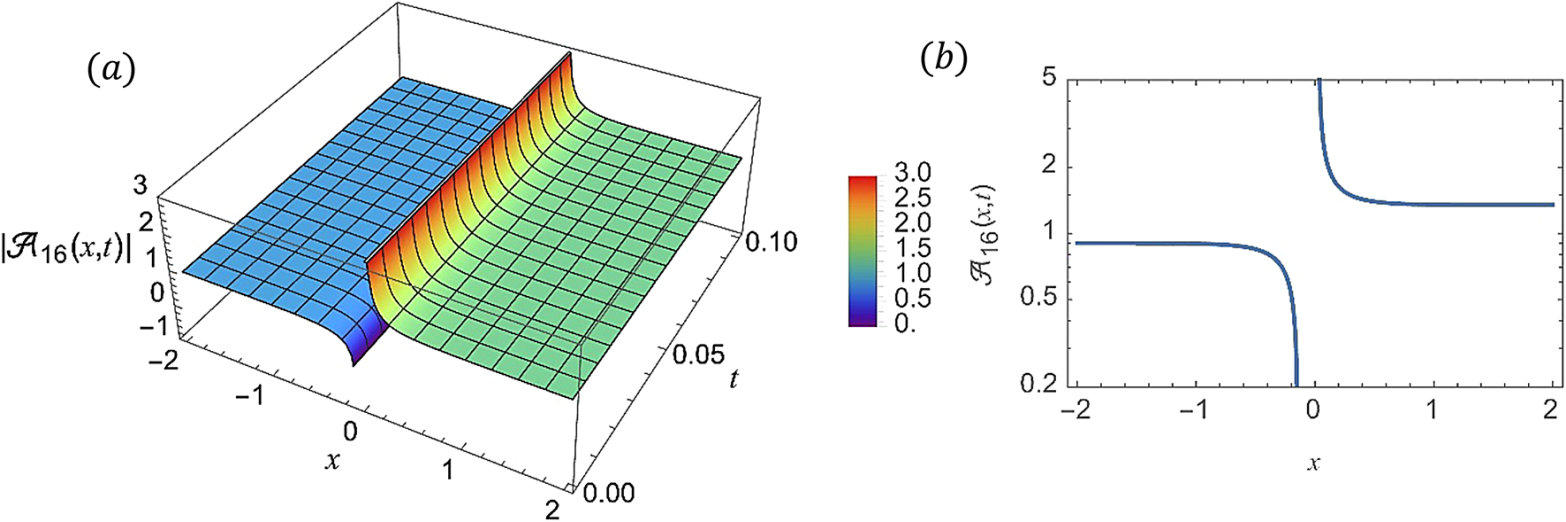
(**a**) 3D graph for $\:\left|{\mathcal{A}}_{16}\left(x,t\right)\right|$ showing the dark singular soliton solution’s physical behavior as a function of * x* and * t* variables. (Eq. (29)). (**b**) 2D graph for $\:\left|{\mathcal{A}}_{16}\left(x,t\right)\right|$ as a function of * x* at $\:t=0.3$.

**Fig. 9:**
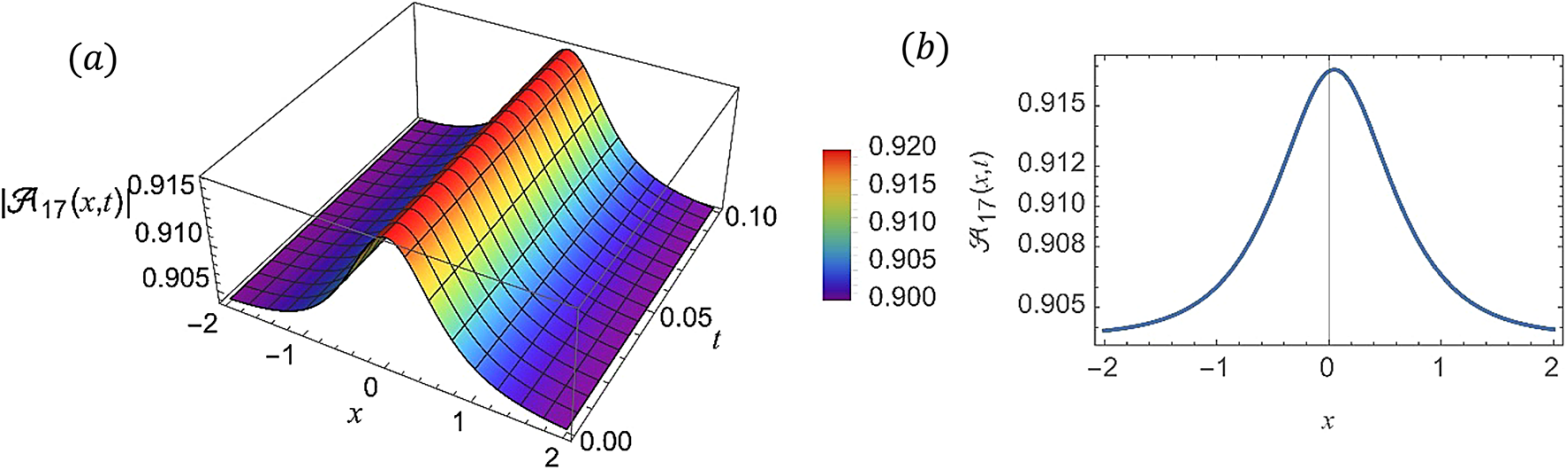
(**a**) 3D graph for $\:\left|{\mathcal{A}}_{17}\left(x,t\right)\right|$ showing the shock wave solution’s physical behavior as a function of *x * and *t * variables. (Eq. (30)). (**b**) 2D graph for $\:\left|{\mathcal{A}}_{17}\left(x,t\right)\right|$ as a function of *x * at $\:t=0.3$.

**Fig. 10:**
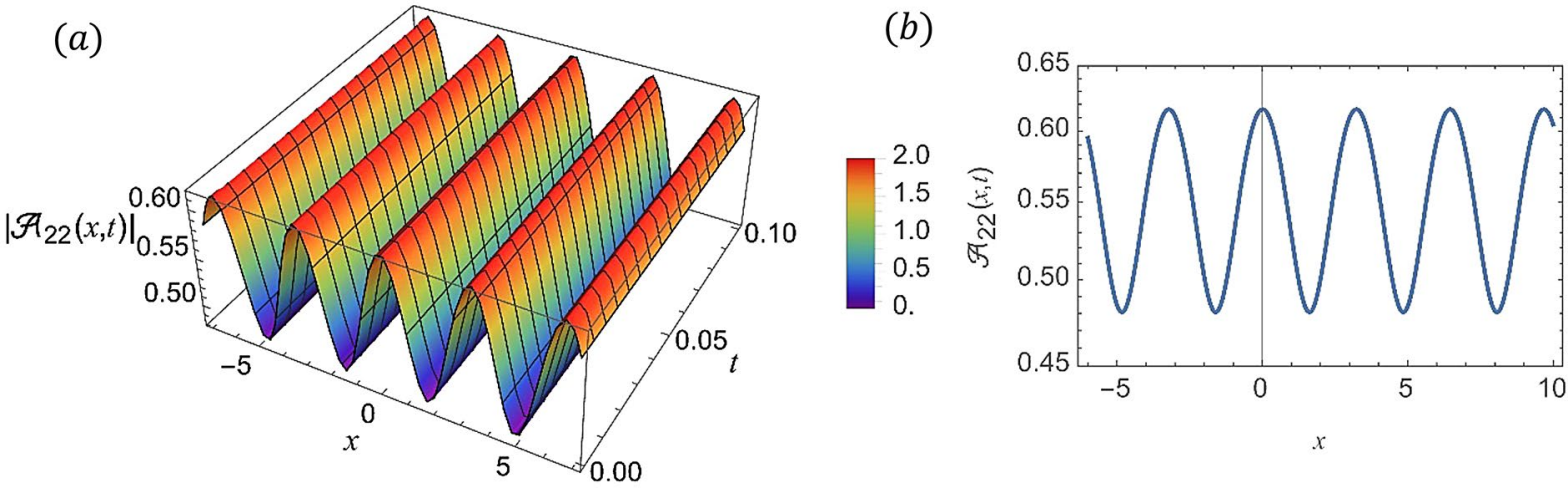
(**a**) 3D graph for $\:\left|{\mathcal{A}}_{22}\left(x,t\right)\right|$ showing the Jacobi Elliptic solution’s physical behavior as a function of * x* and *t * variables. (Eq. (35)). (**b**) 2D graph for $\:\left|{\mathcal{A}}_{22}\left(x,t\right)\right|$ as a function of * x* at $\:t=0.1$.

For various choices of arbitrary parameters inside the proper range space, the modulus of some of these solutions, which stands for the amplitude of the resulting wave, is plotted in the following figures. In these figures, the solutions are presented in both two and three dimensions. Figure 1 shows the bright soliton solution for $\:\left|{\mathcal{A}}_{1}\left(x,t\right)\right|$ (Eq. (14)) with $\:a=0.5,\:{a}_{1}=0.5,\:b=2,\:{b}_{1}=0.2,\:\kappa\:=0.1,\:\omega\:=0.8,\:$and $\:\tau\:=0.1$ with $\:-3\le\:x\le\:3$ and $\:0\le\:t\le\:2$ . Figure 2 shows the singular periodic soliton solution for $\:\left|{\mathcal{A}}_{2}\left(x,t\right)\right|$ (Eq. (15)) with $\:a=0.5,\:{a}_{1}=1.0,\:b=0.2,\:{b}_{1}=0.9,\:\kappa\:=0.1,\:\omega\:=2.0,$ and $\:\tau\:=0.1$ with $\:-2\le\:x\le\:2$ and $\:0\le\:t\le\:2$. Figure 3 presents the hyperbolic soliton solution for $\:\left|{\mathcal{A}}_{3}\left(x,t\right)\right|$ (Eq. (16)) with $\:a=0.5,\:{a}_{1}=1.0,\:b=-0.9,\:{b}_{1}=0.2,\:\kappa\:=0.1,\:\omega\:=2.0,\:\tau\:=0.1,\:{{\varrho}}_{4}=-0.3$ and $\:{\alpha\:}_{-1}=0.7$ with $\:-3\le\:x\le\:3$ and $\:0\le\:t\le\:0.5$. Figure 4 displays the singular bright combo soliton solution for $\:\left|{\mathcal{A}}_{4}\left(x,t\right)\right|$ (Eq. (17)) with $\:a=0.5,\:{a}_{1}=1.0,\:b=0.9,\:{b}_{1}=-0.2,\:\kappa\:=1.0,\:\omega\:=2.0,\:\tau\:=0.1,\:{{\varrho}}_{2}=-1.0$ and $\:{{\varrho}}_{4}=-0.3$ with $\:0\le\:x\le\:1$ and $\:0\le\:t\le\:0.5$. In Fig. 5 shows the periodic soliton solution for $\:\left|{\mathcal{A}}_{11}\left(x,t\right)\right|$ (Eq. (24)) with $\:a=0.5,\:{a}_{1}=0.4,\:b=4.0,\:{b}_{1}=1.0,\:\kappa\:=1.0,\:\omega\:=-0.2,\:\tau\:=0.1,\:$and $\:{\alpha\:}_{0}=0.1$ with $\:-4\le\:x\le\:4$ and $\:0\le\:t\le\:0.1$. Figure 6 shows the plane wave solution for $\:\left|{\mathcal{A}}_{14}\left(x,t\right)\right|$ (Eq. (27)) with $\:a=-0.5,\:{a}_{1}=0.4,\:b=4.0,\:{b}_{1}=0.1,\:\kappa\:=1.0,\:\omega\:=0.2,\:\tau\:=0.1,\:{{\varrho}}_{3}=1.0\:\:$and $\:{{\varrho}}_{4}=-1.0\:$ with $\:-4\le\:x\le\:4$ and $\:0\le\:t\le\:0.1$. Figure 7 shows the dark soliton solution for $\:\left|{\mathcal{A}}_{15}\left(x,t\right)\right|$ (Eq. (28)) with $\:a=-0.5,\:{a}_{1}=0.1,\:b=4.0,\:{b}_{1}=0.1,\:\kappa\:=1.0,\:\omega\:=3.0,\:\tau\:=0.1,\:$and $\:{{\varrho}}_{3}=-1.0$ with $\:-2\le\:x\le\:5$ and $\:0\le\:t\le\:0.1$. Figure 8 shows the singular soliton solution for $\:\left|{\mathcal{A}}_{16}\left(x,t\right)\right|$ (Eq. (29)) with $\:a=-0.5,\:{a}_{1}=0.1,\:b=4.0,\:{b}_{1}=0.1,\:\kappa\:=1.0,\:\omega\:=3.0,\:\tau\:=0.1,\:$and $\:{{\varrho}}_{3}=1.0$ with $\:-2\le\:x\le\:2$ and $\:0\le\:t\le\:0.1$. Figure 9 displays the shock wave solution for $\:\left|{\mathcal{A}}_{17}\left(x,t\right)\right|$ (Eq. (30)) with $\:a=-0.5,\:{a}_{1}=0.1,\:b=4.0,\:{b}_{1}=0.1,\:\kappa\:=1.0,\:\omega\:=3.0,\:\tau\:=0.1,\:$and $\:{{\varrho}}_{3}=1.0$ with $\:-2\le\:x\le\:2$ and $\:0\le\:t\le\:0.1$. Figure 10 displays Jacobi Elliptic soliton solution for $\:\left|{\mathcal{A}}_{22}\left(x,t\right)\right|$ (Eq. (35)) with $\:a=10,\:{a}_{1}=0.1,\:b=7.0,\:{b}_{1}=2.0,\:\kappa\:=1.0,\:\omega\:=3.0,\:\tau\:=0.1,\:\mu\:=0.1\:$and $\:{{\varrho}}_{0}=1.0$with $\:-7\le\:x\le\:7$ and $\:0\le\:t\le\:0.1$.

## Conclusions

This work employed a modified extended mapping technique to obtain precise mathematical formulas for describing various wave patterns generated by the QpGI equation that models the behavior of solitonic and other waves. The approach proved to be highly effective in resolving the intricate problem at hand. The found wave solutions can be applied to a wide range of physical problems under various conditions. These solutions include different patterns such as shocks, singular, periodic waves, and others. These solutions are characterized by stability, adaptability, and the capacity for long-distance travel, making them potentially valuable for addressing complex phenomena in science and engineering. To enhance understanding, 3D and 2D plots were generated to visualize the physical attributes of several solutions. In future research, we hope that the modified extended mapping method will play a key role in solving a variety of non-linear partial differential equations. The research could be extended to solve other types of equations using the modified extended mapping technique. Researchers could also study the stability and behavior of the solutions found, explore their potential applications in fields like optical communication, and improve the modified extended mapping technique to find more soliton solutions.

## Data Availability

All data generated or analyzed during this study are included in this published article.
